# Supplementary material for: Quantification of long-term doxorubicin response dynamics in breast cancer cell lines to direct treatment schedules
Source: PLoS Comput Biol. 2022 Mar 31;18(3):e1009104. doi: 10.1371/journal.pcbi.1009104 (PMC9004764; doi:10.1371/journal.pcbi.1009104)
Supplement: S1 File — Text A. Model identifiability results. Table A. Model assumptions used to generate simulated data sets. Table B. Parameter distributions for simulated data sets. Figure A. Model performance on data with no proliferation delay. Figure B. Model performance on data with a proliferation delay. Figure C. Model performance with fixed vs. calibrated carrying capacity. Figure D. Model performance on matched and mismatched death delay assumptions. Table C. Model performance summarized via PCC. Text B. Constraining the model calibration. Text C. Structure of model preferences. Figure E. Selection of model 1 over model 2. Figure F. Selection of model 1 over model 3. Text D. Details of Model Validation Results. Table D. Leave-one-out validation of model 1 calibration results. Text E. Processing Definition Optimization in the Incucyte Zoom. (PDF) [file pcbi.1009104.s001.pdf]

Supporting Information

"Quantification of long-term doxorubicin response dynamics in breast cancer cell lines to direct treatment schedules"

Authors: Grant R. Howard, Tyler A. Jost, Thomas E. Yankeelov, Amy Brock

Corresponding author: Amy Brock
Email: amy.brock@utexas.edu

Supporting Information Table of Contents

Text A. Model identifiability results

Table A. Model assumptions used to generate simulated data sets.

Table B. Parameter distributions for simulated data sets.

Figure A. Model performance on data with no proliferation delay.

Figure B. Model performance on data with a proliferation delay.

Figure C. Model performance with fixed vs. calibrated carrying capacity.

Figure D. Model performance on matched and mismatched death delay assumptions.

Table C. Model performance summarized via PCC.

Text B. Constraining the model calibration

Text C. Structure of model preferences

Figure E. Selection of model 1 over model 2.

Figure F. Selection of model 1 over model 3.

Text D. Details of Model Validation Results

Table D. Leave-one-out validation of model 1 calibration results.

Text E. Processing Definition Optimization in the Incucyte Zoom

Text A. Model identifiability results

Without *a priori* knowledge of the underlying process, it is difficult to assess the utility of model calibration, which indirectly quantifies parameters such as resistant fraction. Here we approach this problem by generating simulated data sets based on six sets of assumptions about that underlying process. This model family includes three sets of assumptions concerning the form of k, which are described as models 1, 2, and 3; independently, t_r can be zero or non-zero. We test the ability of the modeling framework to correctly characterize the process and to accurately extract parameter values in these cases where we know the underlying ground truth and can quantitatively assess the modeling framework's accuracy.

Data set	Proliferation Delay	Death Delay
A	$t_r \geq 0$	Exponential (Model 1)
B	$t_r \geq 0$	Linear (Model 2)
C	$t_r \geq 0$	None (Model 3)
D	$t_r = 0$	Exponential (Model 1)
E	$t_r = 0$	Linear (Model 2)
F	$t_r = 0$	None (Model 3)

Table A. Model assumptions used to generate simulated data sets. Simulated data sets were generated representing the six possible combinations of assumptions about delays on cell proliferation and cell death following drug exposure.

Each data set consists of 1000 randomly generated parameter sets and 1000 cell number curves generated using the parameter sets described in **Tables A** and **B**. The parameters were generated from distributions based on reasonable physiological assumptions: f_r was selected from a uniform distribution ($0 \leq f_r \leq 1$) in order to fully explore the possible range, while other parameters were selected from normal distributions. Each parameter was bounded at 0.

Data set	A (μ, σ)	B (μ, σ)	C (μ, σ)	D (μ, σ)	E (μ, σ)	F (μ, σ)
t_r	200, 100	200, 100	200, 100	-	-	-
g_r	0.025, 0.005	0.025, 0.005	0.025, 0.005	0.025, 0.005	0.025, 0.005	0.025, 0.005
k_d	0.005, 0.0015	0.005, 0.0015	0.005, 0.0015	0.005, 0.0015	0.005, 0.0015	0.005, 0.0015
t_d	0.02, 0.005	72, 24	-	0.02, 0.005	72, 24	-
N_0	5000, 1000	5000, 1000	5000, 1000	5000, 1000	5000, 1000	5000, 1000
N_{max}	60000, 5000	60000, 5000	60000, 5000	60000, 5000	60000, 5000	60000, 5000
g_0	0.025, 0.002	0.025, 0.002	-	0.025, 0.002	0.025, 0.002	-

Table B. Parameter distributions for simulated data sets. Simulated data set parameter distributions were used to generate data sets A-F. Values are given as mean, standard deviation for each parameter; "-" indicates that the parameter is not present in the model in question

Each of the 6000 total cell number curves was used to calibrate each of the 18 models described on **Table A**. The 18 models stem from these 6 sets of assumptions about the underlying ground truth, along with two additional tests regarding the effectiveness of calibrating the model parameters t_r and N_{max}. Testing whether t_r should be calibrated adds 3 models rather than doubling the number because models 7, 8, and 9 (and corresponding data sets D, E, and F) already assume $t_r = 0$ and therefore do not require further testing. Testing whether N_{max} can be fixed rather than calibrated then doubles the number of models which must be tested to 18.

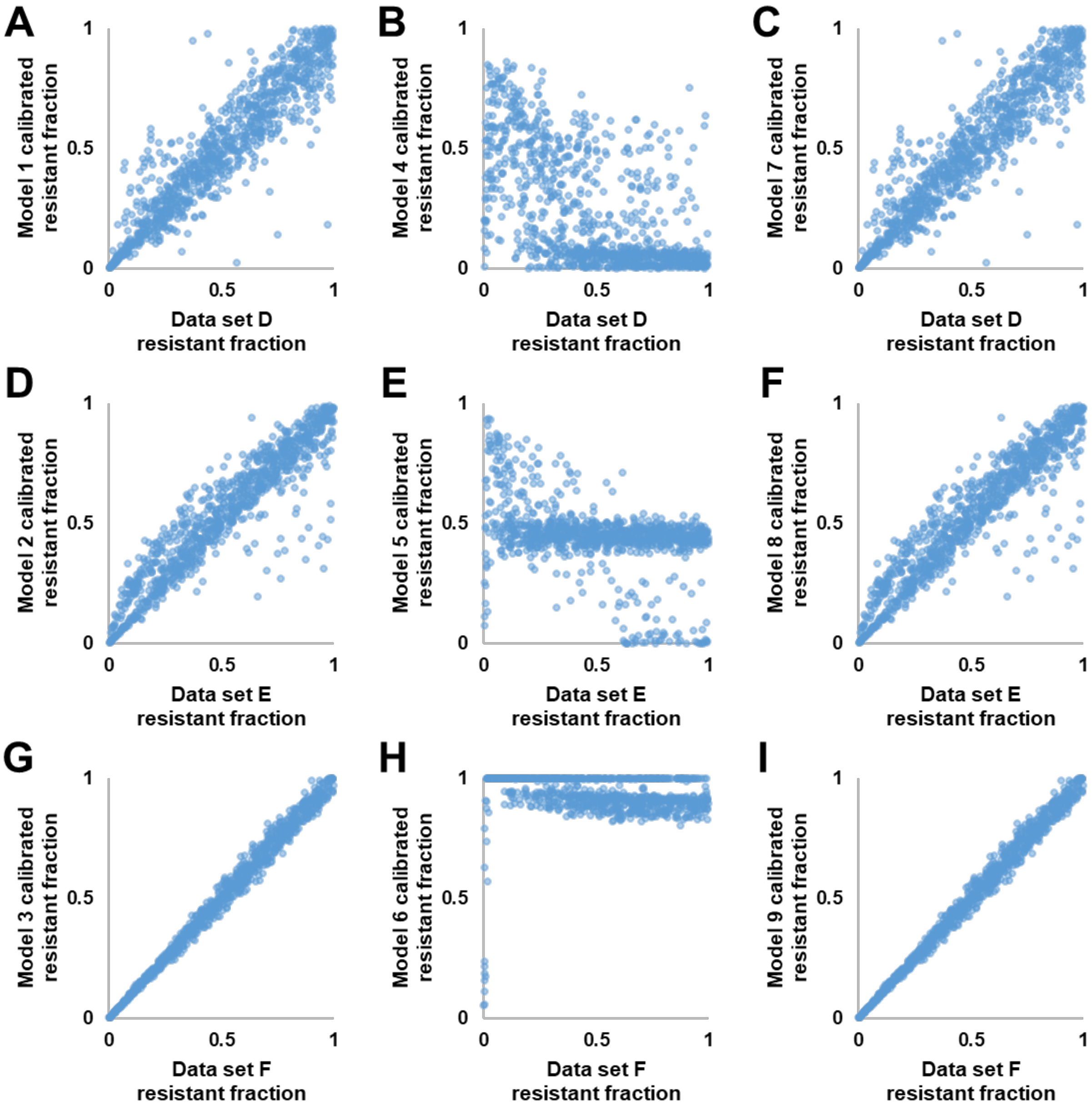

Figure A. Model performance on data with no proliferation delay. Model performance when $t_r = 0$ is tested for models 1-9 by calibrating data generated from parameter sets D, E, and F where $t_r=0$. Each point represents the resistant fraction parameter value extracted from one cell number curve; the closer to the x = y diagonal, the more accurate the extracted parameter value.

When data generated from parameter sets D, E, and F (where $t_r=0$) is used to calibrate models 1-9 (**Fig A**), models 4, 5, and 6 are unable to extract accurate parameter values. Models 1 and 7, models 2 and 8, and models 3 and 9 are equivalent to each other under these circumstances and perform identically. In each case, the model was used to extract parameter values from a matching data set in the handling of the sensitive cell death delay.

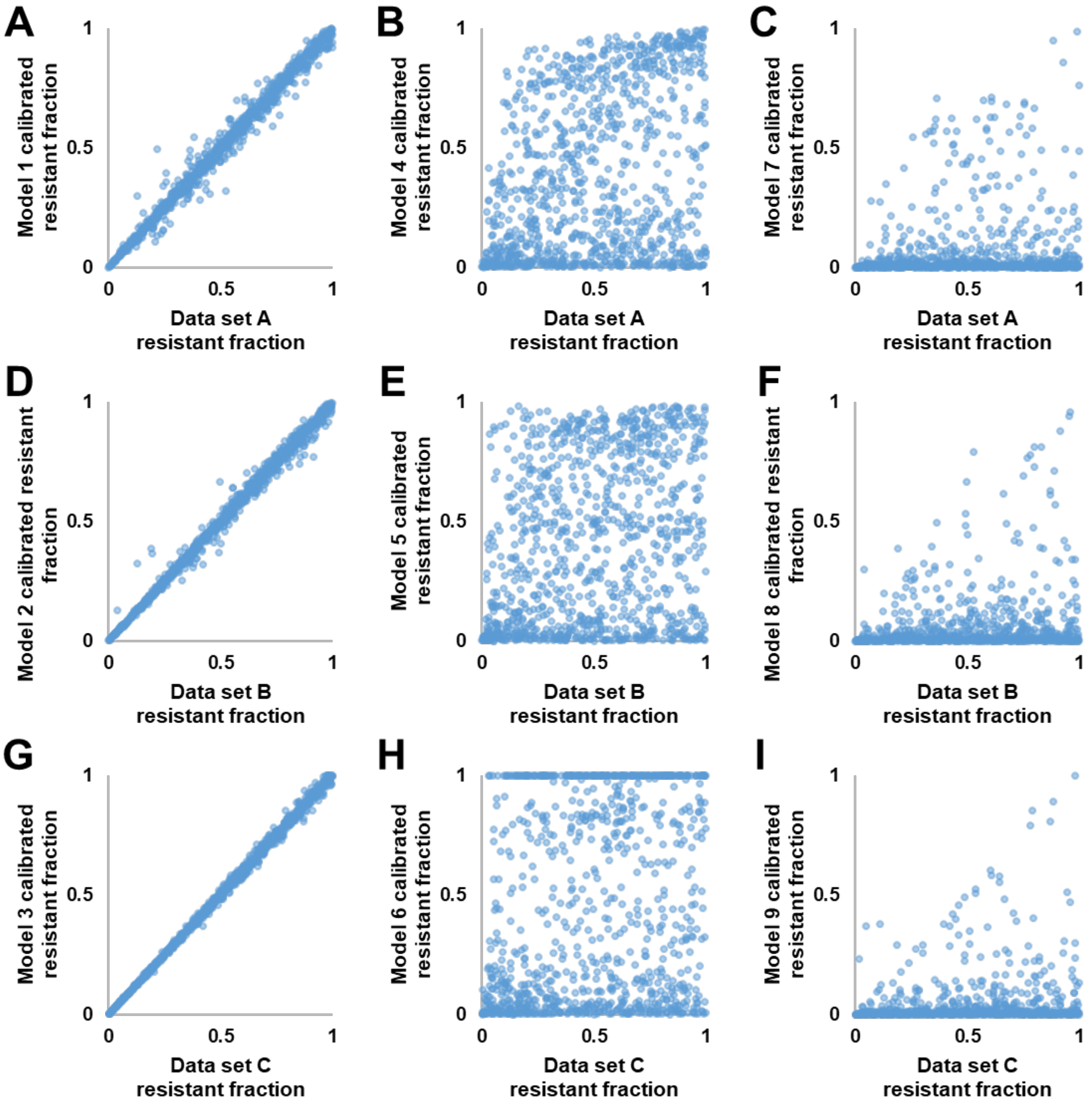

Figure B. Model performance on data with a proliferation delay. Model performance when $t_r \geq 0$ is tested for models 1-9 by calibrating data generated from parameter sets A, B, and C where $t_r \geq 0$. Each point represents the resistant fraction parameter value extracted from one cell number curve; the closer to the $x = y$ diagonal, the more accurate the extracted parameter value.

When data generated from parameter sets A, B, and C (where $t_r \geq 0$) is used to calibrate models 1-9 (**Fig B**), models 4-9 are not capable of extracting accurate parameter values. Once again, in each case the data displayed is for a model used to extract parameter values from the data set with matching handling of sensitive cell death. Combined with **Fig A**, these results prompt the conclusion that models 4-6 never perform acceptably, models 7-9 extract parameter values reasonably well when used on data where $t_r=0$ but do not perform well when used on data where $t_r \geq 0$, and models 1-3 perform suitably under both conditions. Because models 4-6 were unable to accurately calibrate values of t_r even when used with simulated data generated based on identical

assumptions, we conclude that it is not possible to extract accurate values of t_r from the cell number curve alone through computational analysis with our framework; t_r must instead be measured manually. If t_r were found to be zero when manually quantified, models 1-3 and 7-9 would be equivalent in terms of analysis, but models 7-9 would be preferred for presentation due to their lower complexity; if t_r were found to be non-zero (as is indeed the case), models 7-9 would be insufficient and models 1-3 preferred.

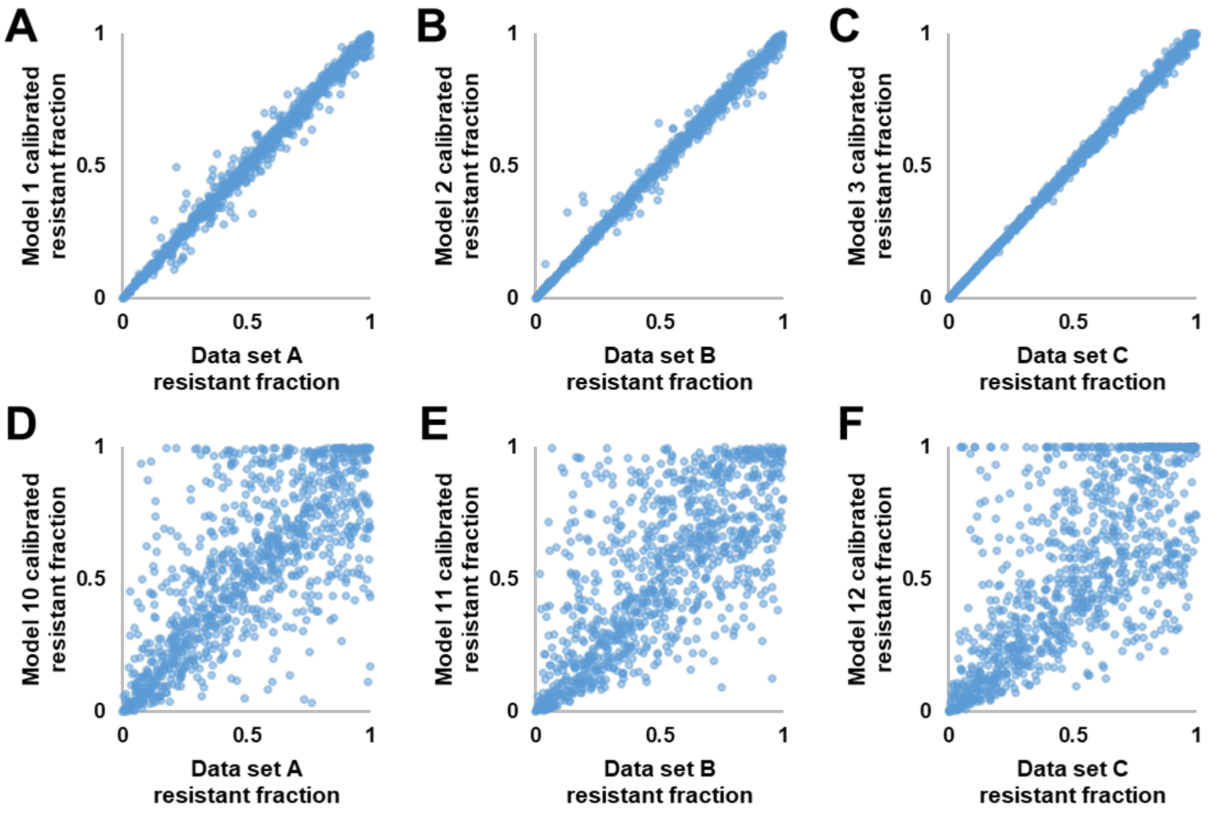

Figure C. Model performance with fixed vs. calibrated carrying capacity. The necessity of calibrating N_{max}, the carrying capacity for logistic growth, is tested by calibrating models 1-3 and 10-12 with their respective matching data sets. Each point represents the resistant fraction parameter value extracted from one cell number curve; the closer to the x = y diagonal, the more accurate the extracted parameter value is.

To test whether the carrying capacity, N_{max}, must be calibrated or whether it could be fixed, we compared the performance of models 1-3 and their matching models 10-12, which are identical other than in the handling of N_{max}. Models 1-3 calibrate N_{max} as a parameter, while models 10-12 use a fixed value. We are testing these models on simulated data for which we know the actual mean carrying capacity and we use the mean of that distribution as the fixed value for models 10-12. Each model was used to calibrate the data set generated under matching assumptions for t_r and k – data set A for models 1 and 10, data set B for models 2 and 11, and data set C for models 3 and 12 (**Fig C**). Models 10-12 exhibit dramatically degraded ability to extract accurate parameter values compared to models 1-3; further, in these simulated data sets we know the true mean carrying capacity – in actual experimental data there is some variation based on experimental conditions. Based on the inability of models 10-12 to extract accurate parameter values even under ideal conditions, we conclude that N_{max} cannot be given a fixed value, and instead must be calibrated.

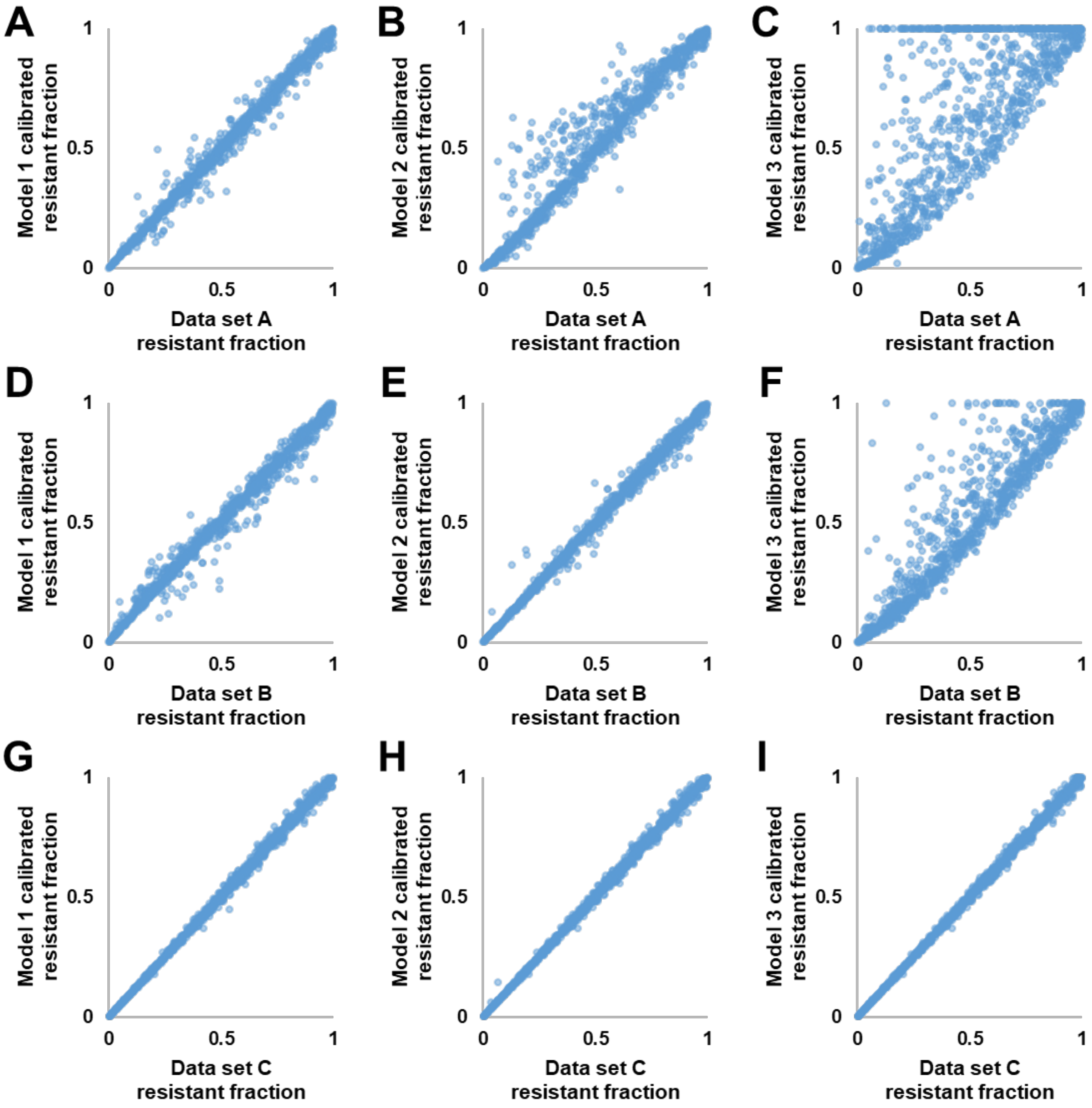

Figure D. Model performance on matched and mismatched death delay assumptions. Model performance under the three forms of death delay is tested by using models 1, 2, and 3 to calibrate simulated data sets A, B, and C. Each point represents the resistant fraction parameter value extracted from one cell number curve; the closer to the x = y diagonal, the more accurate the extracted parameter value.

We then tested our modeling framework for its ability to detect the underlying ground truth among models 1, 2, and 3 by calibrating each of these models to simulated data set A, which was generated based on assumptions matching model 1, simulated data set B, which was generated based on assumptions matching model 2, and simulated data set C, which was generated based on assumptions matching model 3 (**Fig D**). When evaluating the accuracy of extracted parameter values, we found that model 3 does a poor job of extracting parameter values from simulated data sets A and B, which were generated from mismatching sets of assumptions, while models 1 and 2

each perform reasonably well on both data sets A and B, but each model performs better when used on the appropriate matching data set (model 1 with data set A, model 2 with data set B). All three models perform similarly well when used to extract parameter values from data set C. These performance differences can be assessed quantitatively using the PCC, with results matching the visual assessments laid out in **Fig A-D** (**Table C**).

	A	B	C	D	E	F
1	**0.994**	0.992	0.999	0.931	0.903	0.984
2	0.968	**0.997**	0.999	0.846	0.937	0.969
3	0.748	0.935	**0.999**	0.693	0.853	0.996
4	**0.342**	0.266	0.218	-0.612	-0.671	-0.652
5	0.334	**0.277**	0.211	-0.370	-0.408	-0.449
6	0.194	0.241	**0.245**	-0.177	-0.203	-0.154
7	0.123	0.200	0.179	**0.931**	0.903	0.984
8	0.063	0.189	0.184	0.846	**0.937**	0.969
9	-0.066	0.139	0.141	0.693	0.853	**0.996**
10	**0.741**	0.671	0.702	0.228	0.259	0.264
11	0.784	**0.724**	0.734	0.237	0.291	0.237
12	0.532	0.599	**0.706**	0.471	0.609	0.683
13	**0.272**	0.222	0.186	-0.608	-0.658	-0.662
14	0.298	**0.265**	0.189	-0.370	-0.434	-0.471
15	0.200	0.253	**0.235**	0.201	0.179	0.160
16	0.030	0.083	0.134	**0.228**	0.259	0.264
17	0.024	0.092	0.143	0.237	**0.291**	0.237
18	-0.047	0.076	0.129	0.471	0.609	**0.683**

Table C. Model performance summarized via PCC. Model performance evaluated via Pearson's Correlation Coefficient (PCC) between ground truth values of f_r and extracted values of f_r for each possible combination of model and simulated data set. Combinations of a model and data set which match in underlying ground truth assumptions are designated with bold text.

This evaluation of the accuracy of the extracted parameter values demonstrates that extracted parameter values are more accurate when obtained from a model which more closely resembles the underlying ground truth of the process. It also demonstrates that parameter values extracted by our modeling framework are acceptably accurate if the model matches the underlying ground truth (PCCs of 0.994, 0.997, and 0.999 for models 1, 2, and 3 respectively), provided models 1-3 are used in cases where t_r is non-zero, or models 7-9 in cases where t_r can be verified to be 0. These parameter values do not speak to the accurate identification of which model to use; model selection was performed via AIC, and the results conveyed in the main text. To summarize, the AIC correctly selects model 1 for 87% of simulated cell number curves in data set A, model 2 for 84% of simulated cell number curves in data set B, and model 3 in 97% of simulated cell number curves in data set C. This level of accuracy is sufficient to provide strong overall evidence when considering a large data set in total. Additionally, we must keep in mind that these models represent a simplification of the underlying processes; the phenomena we are measuring do not correspond exactly to one model or the other. The selection of one model over the other simply indicates that the measured behavior is better recapitulated by that model under the experimental conditions.

Text B. Constraining the model calibration

One of the key steps in model calibration is properly constraining the parameter space to ensure that the optimum solutions identified by the modeling framework will be physiologically meaningful.

Resistant fraction, f_r

The resistant fraction is allowed to vary from zero to one in all calibrations.

$$0 \leq f_r \leq 1$$

Relapse growth rate, g_r

The relapse growth rate must be constrained with a minimum value in order to require the modeling framework to meaningfully fit f_r. Without such a constraint, in cases where growth is not detected by the end of the experiment, the framework could not distinguish between a solution with a resistant fraction of 0 accompanied by an arbitrary value of g_r and a solution with a growth rate of 0 and an arbitrary value for f_r. The minimum value for g_r was set at $0.003 \frac{cells}{cell*hour}$ based on the practical limit of detectability within the time frame of the experiment. Values of g_r fit at that minimum value of 0.003 should be interpreted to indicate that the growth rate is $0.003 \frac{cells}{cell*hour}$ *or less*, with the experimental setup insufficiently sensitive to limit this more precisely.

A maximum constraint was also placed on g_r, because in cases where f_r approaches 0, the solution is not sensitive to the value of g_r – g_r is arbitrary. A value of $0.1 \frac{cells}{cell*hour}$ was selected for this constraint; this value is more than double the maximum proliferation rate actually observed for any of the cell lines used in this work, so again any value fit at the limit of $0.1 \frac{cells}{cell*hour}$ should be interpreted as indicating that g_r could not be meaningfully fit for that replicate culture.

Sensitive cell death rate, k_d

Maximum and minimum constraints were placed on k_d, such that $0.0005 \frac{cells}{cell*hour} \leq k_d \leq 0.05 \frac{cells}{cell*hour}$. The minimum constraint is based on the limits of detectability over the time frame of the experiment. The maximum constraint was set at an order of magnitude higher than that typically observed in cultures receiving the highest doses of doxorubicin; in such cultures, the death rate can be fit with high confidence due to the prolonged duration of the population decline. While it is possible that such cultures do not exhibit the maximum death rate theoretically possible, we believe it is unlikely that we would observe a death rate more than an order of magnitude higher in this set of experiments. Values of k_d fit at the constraints should be interpreted to indicate that the data for that particular replicate is insufficient to accurately determine k_d.

Sensitive cell death delay half-life, t_d

The sensitive cell death delay half-life, t_d, is constrained with a minimum value of 0.3 hours in all data sets. This minimum value allows model 1 to recapitulate the performance of model 3, which has no time delay on cell death, when appropriate for the data. Values fit at this minimum can be interpreted to suggest that any time delay on cell death is not detectable.

The maximum constraint for t_d is varied based on the time over which the data being calibrated was collected. Because the data for each replicate culture is truncated, either based on the maximum cell number or on data reliability, data sets can vary in duration from approximately 90 hours in some untreated controls to over 600 hours for the highest impact drug exposures. Long data sets allow the sensitivity to detect longer delays on death – in a short data set, it is not possible

to distinguish between cell death with a long delay, and cell survival, so we are limited to detecting cell death that occurs with a shorter delay. The maximum constraint for t_d was varied such that $t_d \leq \frac{t_{final}}{6}$, where t_{final} represents the time of the last data point calibrated for the particular replicate.

Carrying capacity, N_{max}

In data sets where net regrowth is observed for a relatively short amount of time, or where the total cell number is very low over the course of the experiment, the ability to accurately determine the carrying capacity is degraded. Carrying capacity was therefore constrained such that $5{,}000 \leq N_{max} \leq 120{,}000$. These constraints are necessary, because the model fitting can otherwise substitute an arbitrarily low carrying capacity for accurately fitting k_d, and an arbitrarily high carrying capacity can decrease sensitivity to accurately fit f_r and g_r. These constraints were set to be outside of the limits at which we have observed carrying capacity in experiments with sufficient regrowth to calibrate carrying capacity accurately; in replicates in which carrying capacity is calibrated at these limits, the interpretation should be that the data is insufficient to precisely determine carrying capacity.

Text C. Structure of model preferences

While model 1 had the best performance across the range of conditions explored in these experiments, model 2 was preferred in 19.9% of replicate cultures, and model 3 was preferred in 21.1% of cultures. Additionally, the preference for model 1 in 59.0% of replicate cultures is somewhat lower than the 87% identification in simulated data set A, suggesting that the match between model 1 and the underlying biology is somewhat lower than the match between model 1 and the underlying ground truth of data set A. Analysis of these model preferences in greater depth provides additional context for the overall assessment that model 1 is best.

In the 137 replicate cultures for which model 3 was selected as optimal, the magnitude of the ΔAIC between model 3 and either model 1 or 2 was approximately 2, which results simply from the difference in the number of parameters between the less complex model 3 and the more complex models 1 and 2. These data indicate that even in cases where model 3 is ideal, models 1 and 2 converge to the same solution as model 3.

In 21.1% of replicate cultures model 3 was found to be optimal, indicating that there is no detectable delay in sensitive cell death in those cultures, while in 78.9% of replicate cultures, inclusion of a delay on cell death improved model performance even after accounting for the added complexity. In the subset of cultures which did not exhibit a delay in sensitive cell death, all three models converged to identical solutions. Within the subset of cultures where a model incorporating a delay on sensitive cell death was selected, a model representing the delay as an exponential decay (model 1) performed optimally 75% of the time, while a model representing the delay as linear (model 2) performed optimally 25% of the time. Model 1 was therefore selected as the most interesting for further analysis and discussion, due to its overall performance over the range of conditions tested. Although model 2 was not further explored in this manuscript, the substantial minority of cases in which it is selected suggests that the type of process described by model 2 plays a role under some conditions, and exploration of the exact nature of the tradeoffs between these models may be a fruitful area for further inquiry.

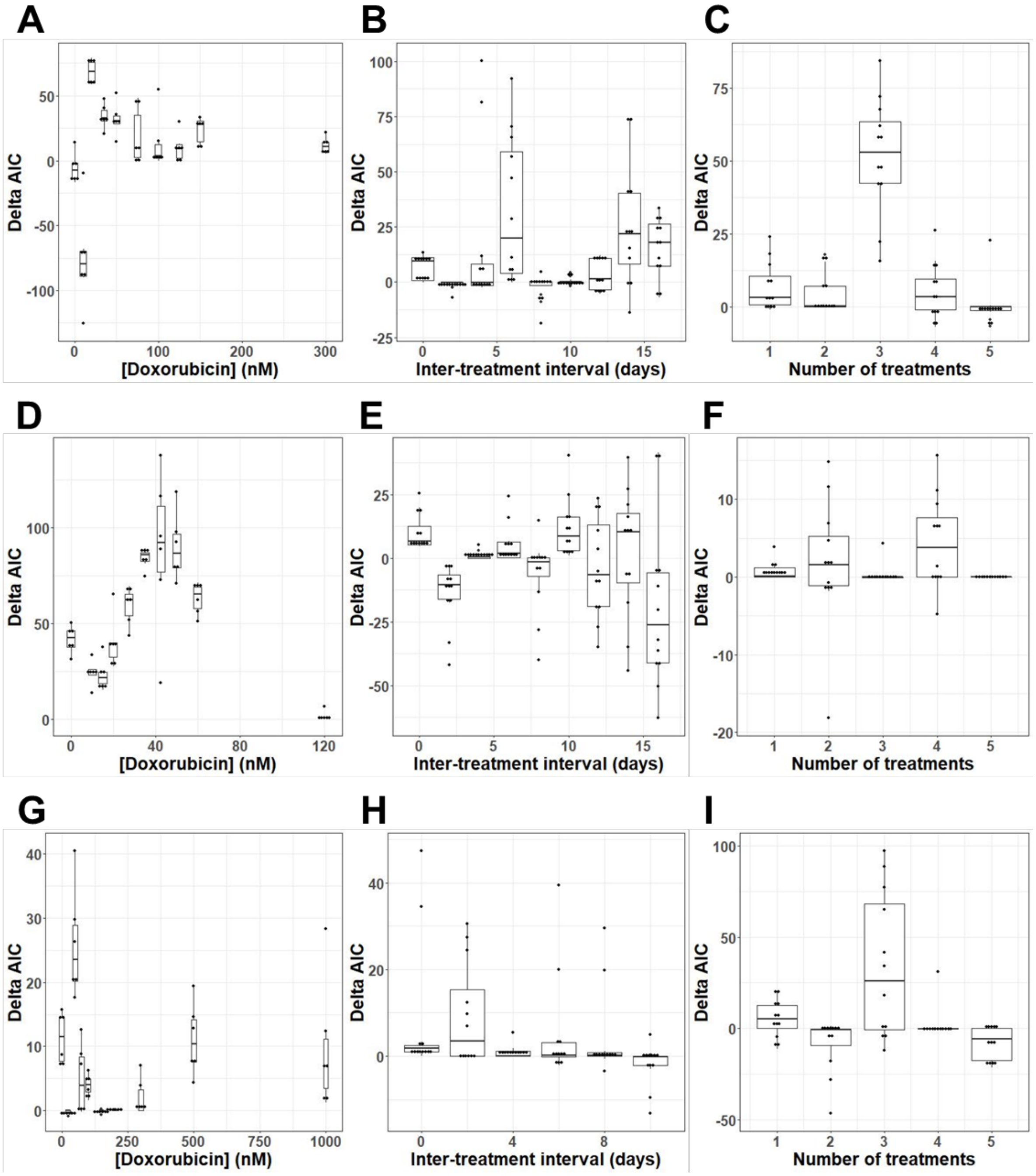

Figure E. Selection of model 1 over model 2. The Akaike Information Criterion is evaluated to quantify the preference for model 1 over model 2 in the MCF7, BT474, and MDA-MB-231 cell lines as doxorubicin concentration varies in the MCF7 cell line (A), the BT474 cell line (D), and the MDA-MB-231 cell line (G), as the interval between two 24 hour doxorubicin exposures varies at 75 nM in the MCF7 cell line (B), at 35 nM in the BT474 cell line (E), and at 200 nM in the MDA-MB-231 cell line (H), and as the number of sequential 24 hour doxorubicin exposures varies at a two day interval and 75 nM in the MCF7 cell line (C), at a zero day interval (continuous exposure) and 35 nM in the BT474 cell line (F), and at a two day interval and 200 nM in the MDA-MB-231 cell line (I). Positive values indicate preference for model 1, while negative values indicate preference for model 2.

Model 1 has better overall performance than model 2, but many experimental groups have mixed preferences, with model 1 performing best on some replicates and model 2 performing best on others (**Fig E**). When drug concentration is varied, all three cell lines consistently prefer model 1, with the exception of the lowest doses in the MCF7 cell line; at these lowest doses variation in the cell death parameters has little effect on the resulting predicted cell number curve, resulting in the modeling framework having low sensitivity to accurately quantify cell death parameters under these circumstances. When the interval between drug exposures is varied, the MCF7 and MDA-MB-231 cell lines tend to show similar performance between the models (ΔAIC near 0), with replicates tending to prefer model 1 when there is a significant difference; in the BT474 cell line, the opposite is true, with the trend leaning towards model 2. This could result from cell-line specific characteristics, or it could be a result of the slightly lower lethality of the dose used for repeated treatment in the BT474 cell line. When the number of sequential treatments is varied, the trend is again for the performance of the two models to be similar and the ΔAIC to be small, with those replicates that do show significant differences in model performance favoring model 1.

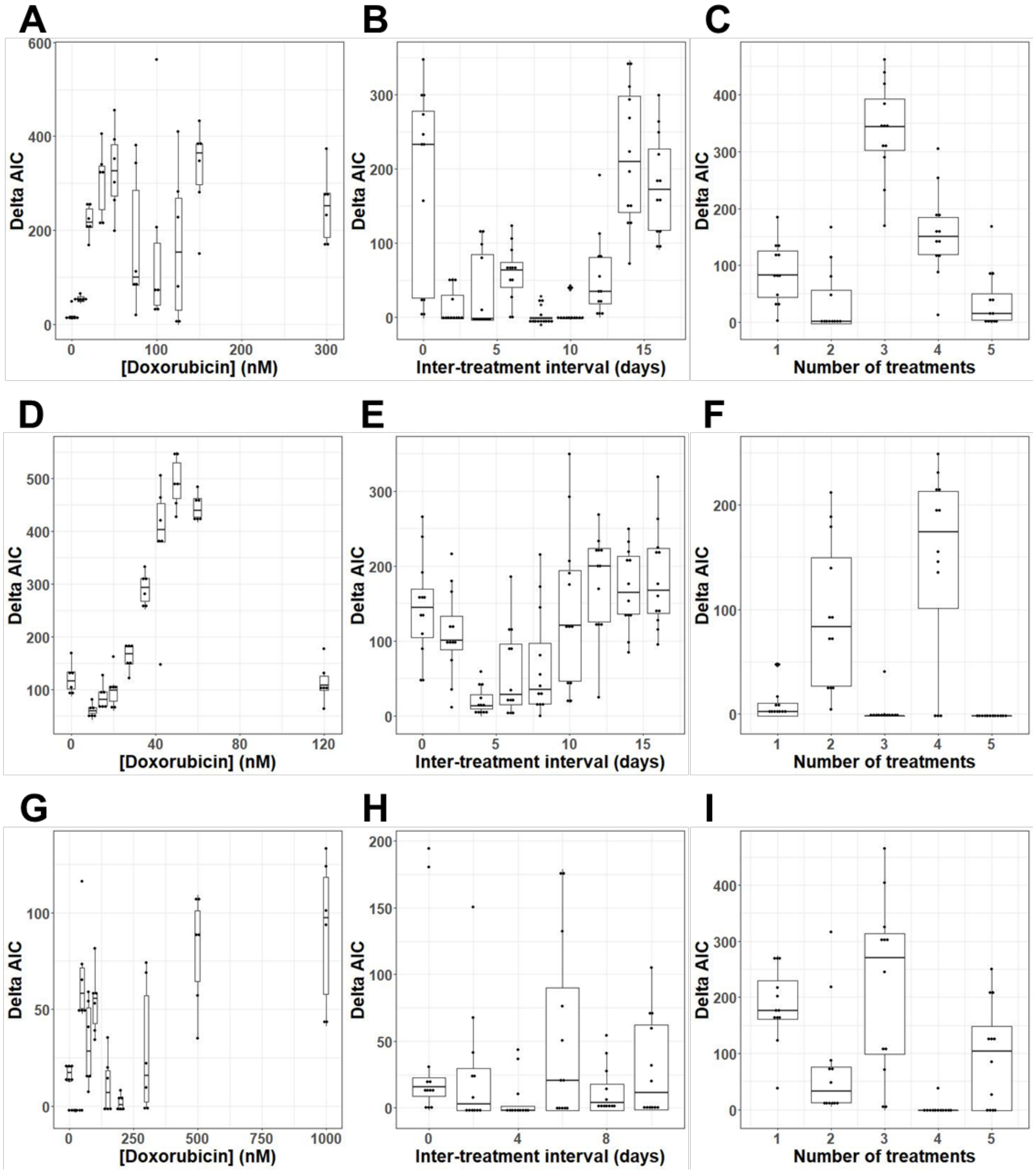

Figure F. Selection of model 1 over model 3. The Akaike Information Criterion is evaluated to quantify the preference for model 1 over model 3 in the MCF7, BT474, and MDA-MB-231 cell lines as doxorubicin concentration varies in the MCF7 cell line (A), the BT474 cell line (D), and the MDA-MB-231 cell line (G), as the interval between two 24 hour doxorubicin exposures varies at 75 nM in the MCF7 cell line (B), at 35 nM in the BT474 cell line (E), and at 200 nM in the MDA-MB-231 cell line (H), and as the number of sequential 24 hour doxorubicin exposures varies at a two day interval and 75 nM in the MCF7 cell line (C), at a zero day interval (continuous exposure) and 35 nM in the BT474 cell line (F), and at a two day interval and 200 nM in the MDA-MB-231 cell line (I). Positive values indicate preference for model 1, while negative values indicate preference for model 3.

Across the range of conditions explored in these experiments, model 1 was widely preferred over model 3 (**Fig F**); this is indicated not only by the direction of preference, but also by magnitude – the AIC values in replicate cultures that prefer model 1 average a much higher magnitude, while those that prefer model 3 always have a ΔAIC of approximately 2, indicating that the two models converged to the same solution and that model 3 is preferred solely due to simplicity.

Text D. Details of Model Validation Results

Overall, 80.6% of the 140,093 data points evaluated in this step fell within the 95% confidence interval generated for that point based on the leave-one-out validation scheme. However, there is significant heterogeneity in model performance across the experimental conditions tested, with the model predictions performing well in some circumstances and poorly in others. The performance for each replicate group tested in the experiment is summarized in **Table D**.

Cell Line	Experiment Type	Replicate Group	Recovering or Dying	Total Points	Points in 95% CI	% 95 CI
MCF7	Dose Response	Untreated Control	Recovering	348	342	98.3%
MCF7	Dose Response	10 nM	Recovering	396	339	85.6%
MCF7	Dose Response	20 nM	Recovering	522	466	89.3%
MCF7	Dose Response	35 nM	Recovering	828	801	96.7%
MCF7	Dose Response	50 nM	Recovering	1170	1009	86.2%
MCF7	Dose Response	75 nM	Recovering	1638	1467	89.6%
MCF7	Dose Response	100 nM	Recovering	1638	1510	92.2%
MCF7	Dose Response	125 nM	Recovering	819	772	94.3%
MCF7	Dose Response	125 nM	Dying	819	218	26.6%
MCF7	Dose Response	150 nM	Recovering	819	751	91.7%
MCF7	Dose Response	150 nM	Dying	819	175	21.4%
MCF7	Dose Response	300 nM	Dying	1638	815	49.8%
MCF7	Inter-Treatment Interval	0 Int	Recovering	1785	1777	99.6%
MCF7	Inter-Treatment Interval	0 Int	Dying	1275	372	29.2%
MCF7	Inter-Treatment Interval	12 Int	Recovering	1968	1956	99.4%
MCF7	Inter-Treatment Interval	14 Int	Recovering	1824	1817	99.6%
MCF7	Inter-Treatment Interval	16 Int	Recovering	1680	1676	99.8%
MCF7	Inter-Treatment Interval	2 Int	Dying	2130	2064	96.9%
MCF7	Inter-Treatment Interval	4 Int	Recovering	1379	1351	98.0%
MCF7	Inter-Treatment Interval	4 Int	Dying	985	966	98.1%
MCF7	Inter-Treatment Interval	6 Int	Recovering	1810	1784	98.6%
MCF7	Inter-Treatment Interval	8 Int	Recovering	1980	1903	96.1%
MCF7	Inter-Treatment Interval	10 Int	Recovering	1812	1669	92.1%
MCF7	Serial Treatment	1 Treatment	Recovering	2904	2539	87.4%
MCF7	Serial Treatment	2 Treatments	Recovering	672	645	96.0%
MCF7	Serial Treatment	2 Treatments	Dying	2016	1773	87.9%
MCF7	Serial Treatment	3 Treatments	Dying	2472	2284	92.4%
MCF7	Serial Treatment	4 Treatments	Dying	2256	2201	97.6%
MCF7	Serial Treatment	5 Treatments	Dying	2040	1913	93.8%
BT474	Dose Response	Untreated Control	Recovering	660	560	84.8%
BT474	Dose Response	10 nM	Recovering	696	595	85.5%
BT474	Dose Response	15 nM	Recovering	1386	1247	90.0%
BT474	Dose Response	20 nM	Recovering	906	755	83.3%
BT474	Dose Response	27.5 nM	Recovering	1038	855	82.4%
BT474	Dose Response	35 nM	Recovering	1128	943	83.6%
BT474	Dose Response	42.5 nM	Recovering	1386	1195	86.2%
BT474	Dose Response	50 nM	Recovering	1386	1183	85.4%
BT474	Dose Response	60 nM	Recovering	1386	1180	85.1%

BT474	Dose Response	120 nM	Dying	1386	605	43.7%
BT474	Serial Treatment	1 Treatment	Recovering	4176	2857	68.4%
BT474	Serial Treatment	2 Treatments	Recovering	5181	4533	87.5%
BT474	Serial Treatment	3 Treatments	Recovering	1392	1184	85.1%
BT474	Serial Treatment	3 Treatments	Dying	4176	999	23.9%
BT474	Serial Treatment	4 Treatments	Dying	4570	3767	82.4%
BT474	Serial Treatment	5 Treatments	Dying	5400	1216	22.5%
BT474	Inter-Treatment Interval	0 Int	Recovering	2574	2166	84.1%
BT474	Inter-Treatment Interval	2 Int	Recovering	2664	2474	92.9%
BT474	Inter-Treatment Interval	4 Int	Recovering	2520	2515	99.8%
BT474	Inter-Treatment Interval	6 Int	Recovering	2376	2345	98.7%
BT474	Inter-Treatment Interval	8 Int	Recovering	2232	2174	97.4%
BT474	Inter-Treatment Interval	10 Int	Recovering	2292	2062	90.0%
BT474	Inter-Treatment Interval	12 Int	Recovering	2148	1886	87.8%
BT474	Inter-Treatment Interval	14 Int	Recovering	2004	1780	88.8%
BT474	Inter-Treatment Interval	16 Int	Recovering	1860	1660	89.2%
MDA-MB-231	Dose Response	Untreated Control	Recovering	384	307	79.9%
MDA-MB-231	Dose Response	25 nM	Recovering	486	457	94.0%
MDA-MB-231	Dose Response	50 nM	Recovering	684	579	84.6%
MDA-MB-231	Dose Response	75 nM	Recovering	684	662	96.8%
MDA-MB-231	Dose Response	100 nM	Recovering	804	647	80.5%
MDA-MB-231	Dose Response	150 nM	Recovering	924	882	95.5%
MDA-MB-231	Dose Response	200 nM	Recovering	1020	964	94.5%
MDA-MB-231	Dose Response	300 nM	Recovering	1194	1078	90.3%
MDA-MB-231	Dose Response	500 nM	Recovering	1188	1054	88.7%
MDA-MB-231	Dose Response	1000 nM	Dying	1188	633	53.3%
MDA-MB-231	Inter-Treatment Interval	0 Int	Recovering	2952	2624	88.9%
MDA-MB-231	Inter-Treatment Interval	2Int	Recovering	1380	1324	95.9%
MDA-MB-231	Inter-Treatment Interval	2 Int	Dying	1380	315	22.8%
MDA-MB-231	Inter-Treatment Interval	4 Int	Recovering	1410	953	67.6%
MDA-MB-231	Inter-Treatment Interval	4 Int	Dying	1410	661	46.9%
MDA-MB-231	Inter-Treatment Interval	6 Int	Recovering	1752	1672	95.4%
MDA-MB-231	Inter-Treatment Interval	6 Int	Dying	876	531	60.6%
MDA-MB-231	Inter-Treatment Interval	8 Int	Recovering	1827	1770	96.9%
MDA-MB-231	Inter-Treatment Interval	8 Int	Dying	609	243	39.9%
MDA-MB-231	Inter-Treatment Interval	10 Int	Recovering	1683	1579	93.8%
MDA-MB-231	Inter-Treatment Interval	10 Int	Dying	561	185	33.0%
MDA-MB-231	Serial Treatment	1 Treatment	Recovering	3396	3150	92.8%
MDA-MB-231	Serial Treatment	2 Treatments	Recovering	4620	3907	84.6%
MDA-MB-231	Serial Treatment	3 Treatments	Recovering	1053	844	80.2%
MDA-MB-231	Serial Treatment	3 Treatments	Dying	3159	2876	91.0%
MDA-MB-231	Serial Treatment	4 Treatments	Dying	3792	2541	67.0%

MDA-MB-231	Serial Treatment	5 Treatments	Dying	3312	2834	85.6%

Table D. Leave-one-out validation of model 1 calibration results. Predictive power of model 1 is analyzed by comparing each cell number curve to a projected distribution created with a leave-one-out approach, and determining what fraction of the data falls within the 95% confidence interval of the distribution.

Overall trends are consistent with those observable in **Table C**, with model 1 performing somewhat better for the MCF7 cell line than for the BT474 and MDA-MB-231 cell lines, and model 1 performing better for cell populations which eventually recover than for those which never recover.

Text E. Processing Definition Optimization in the Incucyte Zoom

Optimal image processing settings vary from experiment to experiment. This results from factors such as differences in cell size and morphology between cell lines, variation in cell size and morphology over the course of an experiment as a result of drug exposure, variation in fluorescent signal brightness (in the experiments presented here, the brightness of the nuclear localized GFP is normally stable in a given cell line under normal growth conditions, but it sometimes changes after drug exposure), and variation in background fluorescence levels. Consequently, we can not give a single value for the optimal parameter settings. Instead we present an optimization procedure. The processing parameters of each experiment were determined as follows:

1. Open the experiment file and generate an image collection. This image collection should include a minimum of three images:
    a. One image shortly after cells have adhered (24-48 hours after cell seeding), allowing characterization of the cells in their initial morphology and size, and at a low cell density.
    b. One image late in the experiment in a well that has grown to 50% confluence or more, allowing characterization of the post treatment morphology and size, and at a high cell density.
    c. One image at the height of drug impact, to allow characterization of any transient changes in cell size and morphology as a result of the drug response. For this initial image collection it is generally best to use an image that is near the center of the range of conditions being tested - a moderate dose in a dose response experiment, a moderate interval in an inter-treatment interval, or a moderate number of drug exposures for a serial treatment experiment.

    You can add additional images if you know that your experiment includes additional qualitatively different conditions. You want the image collection to represent the range of conditions in the data set.
2. Create a new processing definition using this image collection. Alternatively, if you have an existing processing definition for this cell line under similar conditions, you can load the new image collection into your existing processing definition and skip to step 4.
3. Run an initial analysis to visualize the results of the initial parameter values. At this stage, we recommend the following settings:
    a. Uncheck the phase analysis to reduce processing time; cell counting is performed using the green fluorescent image.
    b. In the green channel analysis, the "Parameters" field represents the background subtraction algorithm. We have found the Top Hat method of background subtraction to be optimal in all analyses of whole-well images in 96-well plates. For the time being, leave the radius setting at its default value.
    c. Set a minimum area filter; 10 μm^2 is a good starting point, although you may adjust this downward for cell lines with particularly small nuclei. This excludes single bright pixels and small debris from the count.
    d. Set a maximum eccentricity filter to 0.99. This excludes scratches on the well plate and many imaging artifacts from the count without removing any actual cells.
4. Optimize the Top Hat radius for a single image. Zoom in to several regions of the image and check the quality of the background subtraction. The default settings will often

perform acceptably, but if you see regions with significant background brightness (visually appearing to be on the same order of magnitude as the cell nuclei you want to count), adjust the radius setting up and down slightly to attempt to resolve this. With some settings, you may see imaging artifacts appear in the background as sharp edges in the background fluorescence level. It is particularly important to adjust the radius parameter until these disappear, as they tend to be picked up in the cell count if they are not eliminated at this point. We have found values for the radius between 10 and 80 to be useful, and generally search in this range. During each optimization step, you should zoom in to several regions of the image and make sure that the performance is consistent throughout.

5. Once you have optimized the radius parameter for a single image from your image collection, check the other images in the image collection. Generally a single parameter value will work consistently for all images from a single experiment, but in some cases you will need to iterate through the images, find the range of values that works reasonably well for each, and pick the value that makes the best tradeoff for average quality. In cases where the background subtraction proves particularly difficult, you may choose to implement the optional step 8 to compensate.
6. Optimize the fluorescence Threshold parameter for a single image. Look at the overlay between the green channel and the green mask channel. Manipulate this parameter until the green mask is slightly inside of the edge of the visible green region for a typical cell. Generally you want to set this threshold as high as you can while still capturing the majority of the visibly green area of the nuclei, because a high threshold reduces the effect of background fluorescence and improves counting of tightly clustered cells. The optimal value for this parameter is usually between 0.5 and 2, although it can occasionally vary from as low as 0.3 for especially dim cells to as high as 20 for especially bright cells.
7. Cycle through the other images in your image collection, checking the optimal threshold value. You will need to select the lowest of these threshold values, corresponding to the image in which your cells are least bright, to ensure that counting will work in all images.
8. Optionally, set the Adjust Size parameter to 1 or 2 pixels, and raise the threshold to compensate, going back through steps 6 and 7 with Adjust Size turned on. This can be a useful way to compensate for lower quality in the background subtraction process. By identifying only the brightest cores of cells for counting (using the high threshold) you can bypass a high level of background fluorescence. Using a very high threshold requires you to add in the Adjust Size parameter to avoid dividing single bright cells into multiple regions and multi-counting them. The Adjust Size parameter expands all identified areas outward, which generally results in collapsing these divided cells back into a single counted region. This process is imperfect, and it introduces a small amount of additional error into the cell count. As a result we recommend skipping this unless you find it necessary as a result of high background fluorescence that is resistant to subtraction or dim cells that are close to the background brightness.
9. Optimize the minimum area filter. The minimum area filter can be used to remove debris that is smaller than an actual cell. To optimize this parameter, you should set both a minimum and maximum area filter, and check which marked objects fall within that band. For example, if you have started with a 10 μm^2 filter, you can set the maximum area filter to 20 μm^2, visually scan the objects now marked on the green object mask, and decide if any of them are actually cells by checking the phase contrast image. If few of the marked objects are cells, you can increase the minimum filter to 20 μm^2, increase the maximum to

30 μm^2, and check the next size band. Continue increasing the minimum area filter until you start to lose cells.

10. Cycle through the images in your image collection and ensure that you are not losing a significant number of cells from any of them. You should select a minimum area filter that removes at most a handful of the smallest cells from your image collection.
11. Optimize Edge Sensitivity. Start with the image in your collection with the highest cell density. Zoom in to dense regions where cells are closely clustered, and check whether they are being accurately separated by comparing the green channel and the green object mask. The phase contrast image can be consulted to aid in distinguishing cells. In most cases, good thresholding will facilitate accurate counting with edge sensitivity at the default setting. When cells are clustered particularly tightly, they may overlap enough to interfere with this, and in those cases increasing the edge sensitivity parameter is helpful. The edge sensitivity parameter should be tuned so that it splits clusters accurately without splitting any individual cells if possible, or tuned so that the overcounting and undercounting errors balance each other out if necessary. The cell count is sensitive to changes in the edge sensitivity, so adjustments should be as small as possible. It's generally advisable to keep the edge sensitivity parameter between -10 and +20, and adjustments leaving that range should be made cautiously and checked particularly carefully to make sure that they haven't caused systematic miscounting.
12. Cycle through the images in your image collection and ensure that the cell counts are accurate under the selected edge sensitivity parameters.
13. Set the Whole Well Keep-out. When using whole-well imaging, the wall of the well is usually captured as a bright ring in the fluorescence channel. This large green region will sometimes be counted as hundreds or thousands of cells depending on the variation in the brightness, the threshold in the experiment, etc. To avoid this, the whole well keep-out trims images inward from the edge by the specified number of pixels. You should set this to completely exclude the wall of the well from all images in your image collection, which can generally be done with settings in the range of 0-20 pixels.
14. Save the processing definition and start an analysis job applying it to your data set. This can take up to 24 hours depending on the number of images in your experiment and the ongoing workload on your Incucyte instrument, so if time is a constraint you can start by analyzing a subset of your data. If you take this approach, it is a good idea to include one or two wells from each replicate set rather than one replicate set out of the experiment, so that you can test the processing definition across the range of conditions in your experiment.
15. Once the analysis job is complete, open the analysis job metrics and graph the green object count, which is the metric corresponding to cell number, for your data set. This will allow you to make an initial estimate of the quality of the analysis. Ideally you want to see smooth curves with few discontinuities, and with general trends corresponding to your expectations for the experiment. If you see unexpected discontinuities or regions of some curves with high noise levels, note the time stamps for several discontinuities or noisy regions.
16. Switch to the completed analysis job window. Start by spot checking several images to make sure that the image processing is accurate in the regions where the cell counts are smooth. This is generally the case, but occasionally you will find that there is systematic over or undercounting. To verify that the count is accurate, you should zoom into several

regions of each image and check the overlay between the green object mask and the green channel image. The green object mask should clearly mark each cell nucleus as a single object. It is acceptable for there to be occasional inaccuracies - such as a single cell that is split into multiple objects, or a cluster of cells that are marked as a single object - so long as the inaccuracies are rare (below 1% occurrence) and balanced, with roughly equal undercounting and overcounting. Should you determine that the count is not accurate, select one image which exhibits the inaccuracies and add it to your image collection. It isn't necessary to check every well, or possible to check every image over the course of the experiment, but we recommend checking at least one well at each end of the range of the variable being tested, and for each well checking one early image, one late image, and one midway between.

17. Navigate to the wells and time stamps that correspond to discontinuities or noise in the green object count curve. Check before and after the discontinuity, pick the image that is less accurately counted, and add that image to the image collection. You will often find that there is a specific issue that is common to multiple miscounted regions - for example, the brightness of the cells may change from one image to the next, resulting in a large number of cells falling below the detection threshold. If multiple discontinuities result from the same issue, pick only one of the images to add to your image collection.
18. Return to step 4, and repeat your optimization of the image processing parameters on the expanded image collection. Iterate this process until you are satisfied that the count is as accurate as possible. It will not be possible to remove all discontinuities, as you will find that some discontinuities result from inherent problems with the images. For example, some images may be out of focus, and some discontinuities may result from actual disruption of the cell populations. The iterative process of identifying discontinuities and checking them will clear the correctable issues until the count is as accurate as possible.
